# A Variant of *IL6R* Is Associated with the Recurrence of Atrial Fibrillation after Catheter Ablation in a Chinese Han Population

**DOI:** 10.1371/journal.pone.0099623

**Published:** 2014-06-18

**Authors:** Gang Wu, Mian Cheng, He Huang, Bo Yang, Hong Jiang, Congxin Huang

**Affiliations:** 1 Department of Cardiology, Renmin Hospital of Wuhan University, Wuhan, Hubei, China; 2 Department of Geriatrics, Tongji Hospital, Tongji Medical College, Huazhong University of Science and Technology, Wuhan, Hubei, China; University of Buenos Aires, Faculty of Medicine. Cardiovascular Pathophysiology Institute, Argentina

## Abstract

**Background:**

Recent studies have identified a variant, rs4845625, in the interleukin-6 receptor (IL6R) gene associated with Atrial Fibrillation (AF). Levels of circulating interleukin-6 and other proinflammatory molecules have consistently been associated with a risk for AF and its recurrence after catheter ablation. This study tested the hypothesis that variant rs4845625 is associated with AF recurrence after catheter ablation in a Chinese Han population.

**Methods:**

A total of 278 consecutive patients (mean age 59.4±11.5 years, 43% female) with paroxysmal (36.0%), persistent (59.7%), and permanent (4.3%) AF who underwent catheterablation from 2007–2011, were included in this study. Patients were monitored for 12 months for a recurrence of AF. The SNP rs4845625 was genotyped using high resolution melting analysis.

**Results:**

In our study cohort, an early recurrence of AF (ERAF), defined as a recurrence within the first 4 weeks, was observed in 42.8% of the patients, whereas late recurrence of AF (LRAF) (between 3 and 12 months) occurred in 25.9% of the patients. No significant differences in baseline clinical or echocardiographic characteristics were observed between patients with ERAF and LRAF. In contrast, the presence of the T allele of rs4845625 was associated with an increase in the risk for both ERAF (odds ratio [OR]: 1.84, 95% confidence interval [CI]: 1.31–2.59, p = 4.10×10^−4^) and LRAF (OR: 1.92, 95% CI: 1.30–2.81, p = 0.001). Furthermore, this association was significant after adjustments for age, sex, hypertension, diabetes and other risk factors. No significant relationship between rs4845625 and serum levels of IL6 was observed.

**Conclusions:**

In this study, a variant of the *IL6R* gene, rs4845625, was found confer risk to AF recurrence after catheter ablation in a Chinese Han population. Our findings indicated that the *IL6R* pathway or inflammation may play important rols in the recurrence of AF after catheter ablation.

## Introduction

Atrial fibrillation (AF) is the most common arrhythmia observed in the clinical setting and is an independent risk factor for stroke. Association studies have reported that individuals who carry certain common single-nucleotide polymorphisms (SNPs) in the genes encoding cardiac ion channels, the renin–angiotensin system, or connexin 40 are predisposed to developing AF. Recently, a genome-wide association study identified a variant named rs4845625, which is located in the intron of the *IL6R* gene and is associated with AF [1].

*IL6R* encodes the receptor for interleukin-6, which is a regulator of inflammation and has been reported to be associated with the pathology of AF and a recurrence of AF after catheter ablation. Thus, we examined the relationship between the rs4845625 polymorphism in the *IL6R* gene and AF recurrence after catheter ablation.

## Methods

This retrospective study included 278 patients (mean age 59.4±11.5 years, 43% female) who underwent left atrial catheter ablation for drug-refractory paroxysmal AF (36.0%), persistent AF (59.7%) or permanent AF (4.3%) from 2007–2011 (Table 1). All patients underwent electrocardiography to detect AF recurrences. Of these, 119 patients (42.8%) experienced an early recurrence of atrial fibrillation (ERAF) and 72 patients (25.9%) experienced a late recurrence of atrial fibrillation (LRAF). Diagnoses of AF, lone AF, hypertension (HT) and diabetes were based on standard diagnostic criteria [2], [3], [4]. The study subjects were from the GeneID population, which is a Chinese Han database of clinical data from more than 30,000 Chinese patients and healthy individuals and is used for the identification of susceptibility genes for various cardiovascular diseases [5]. Our study was approved by the Wuhan University institutional review boards on human subject research and conforms to the guidelines set forth by the Declaration of Helsinki. Written informed consent was obtained from the participants, who completed a health questionnaire and provided fasting blood samples.

**Table 1:** Clinical Characteristics of the Study Population.

	ERAF (n = 119)	Without ERAF (n = 159)	P	LRAF (n = 72)	Without LRAF (n = 206)	P
Age (years)*	59.7±11.0	59.0±11.8	0.63	61.3±11.3	58.6±11.8	0.08
Sex, female n (%)	55 (46.2)	64 (40.2)	0.29	32 (44.4)	87 (42.2)	0.78
Hypertension† n (%)	37 (31.1)	56 (35.2)	0.47	24 (33.3)	69 (33.5)	0.98
Diabetes n (%)‡	13 (10.9)	24 (15.1)	0.31	12 (16.7)	25 (12.1)	0.33
Statins n (%)	24 (20.2)	31 (19.5)	0.88	18 (25.0)	47 (22.8)	0.71
ACEI/ARB n (%)	31 (26.1)	51 (32.1)	0.44	24 (33.3)	67 (32.5)	0.90
BB n (%)	23 (19.3)	32 (20.1)	0.87	19 (26.4)	42 (20.4)	0.29
AF category						
Paroxysmal n (%)	45 (37.9)	55 (34.6)	0.58	21 (29.2)	79 (38.3)	0.16
Persistent n (%)	68 (57.1)	98 (61.6)	0.45	49 (68.1)	117 (56.8)	0.09
Permanent n (%)	6 (5.0)	6 (3.8)	0.61	2 (2.8)	10 (3.9)	0.55
Lone AF n (%)	75 (63.0)	117 (73.4)	0.06	54	138	0.21
LAD mm	42±6	43±8	0.66	42±7	42±8	0.71
LVEF %	60±5	61±6	0.41	61±7	61±9	0.51

AF: atrial fibrillation; LAD: left atrial diameter; LVEF: left ventricular ejection fraction; ERAF: early recurrence of atrial fibrillation; LRAF: late recurrence of atrial fibrillation; ACEI: ACE inhibitor; ARB: angiotensin receptor blocker; BB: beta block.

*Age was defined as the time the patient underwent left atrial catheter ablation.

† Hypertension was diagnosed as a blood pressure higher than 140/90 mmHg.

‡ Diabetes was defined as ongoing therapy for diabetes or a fasting plasma glucose level of ≥7.0 mmol/L.

### Catheter Ablation

Left atrial catheter ablation was performed using a previously described approach. In brief, the CARTO mapping system (CARTO, Biosense Webster) was used for nonfluoroscopic 3-dimensional catheter orientation, computed tomographic image integration, and tagging of the ablation sites, with the coronary sinus lead 5/6 serving as a system reference. The ablation was performed with a temperature-controlled, quadripolar, deflectable catheter with an 8-mm tip (Navistar, Biosense Webster). In all patients, circumferential left atrial ablation lines were placed around the antrum of the ipsilateral pulmonary veins (irrigated-tip catheter, pre-selected tip temperature of 50°C, and a maximum power of 30 to 50 W). In patients with persistent or permanent AF, additional linear lesions were added at the left atrial roof, the basal posterior wall, and the left atrial isthmus. Ablations of areas with complex fractionated electrograms were not performed.

After circumferential line placement, voltage and pace mapping along the ablation line were used to identify and close gaps. The isolation of all pulmonary veins with a bidirectional block was verified with a multipolar circular mapping catheter (Lasso) and was defined as the procedural end point.

### Follow-up

Class I and III antiarrhythmic drugs were not reinitiated after ablation. Oral anticoagulation was prescribed for 3 months. All patients received follow-up care in the outpatient clinic for 12 months after the ablation. During this follow-up period, AF was assessed during the first 4 weeks using an event recorder (with routine daily transmissions and additional transmissions for symptoms) and a 24 h Holter monitor. Monitoring was also performed at 3, 6, 9 and 12 months after the ablation. Additional Holter monitoring or event recording was performed for patients with symptoms. In some patients, asymptomatic AF was detected using the information that was received from implanted devices. An AF recurrence was defined as a documented AF episode lasting longer than 30 seconds. An early recurrence of atrial fibrillation (ERAF) was defined as an AF episode during the first 4 weeks after the ablation, similar to previous definitions. This definition was also chosen because an event recorder was available for all patients for this time period. A late recurrence of atrial fibrillation (LRAF) was defined as any AF episode between 3 and 12 months after the ablation. All patients with sustained early recurring AF underwent direct-current cardioversion. Any additional drug administration was left to the discretion of the treating physician.

### Genotyping

DNA was extracted from the patients’ blood samples. The SNP rs4845625 was genotyped using a Rotor-Gene 6000 High Resolution Melt system (Corbett Life Science, Concorde, NSW, Australia) in a 25 µL polymerase chain reaction (PCR) volume containing 0.7 µL of Syto 9 dye, 5 pmol of each primer, 25 ng of genomic DNA, 2.5 µL of 10x PCR buffer with 1.5 mmol/l MgCl_2_, 5 mmol deoxynucleotide triphosphates, and 1 U of Taq polymerase. The forward primer for the High Resolution Melting (HRM) was 5′-tccaaggtgacatagctcgt-3′ and the reverse was 5′-acctgcctctccaccaaaag-3′. One positive control for each genotype (T/T, T/C, and C/C) and one appropriate negative control were included in each run. The positive controls were verified by direct DNA sequence analysis. A call rate of greater than 95% was achieved with this method. Genotypes were confirmed with direct sequencing.

### Serum IL6 Measurement

Serum samples were collected from 48 subjects (16 samples of each genotype CC, CT and TT). All samples were processed by centrifugation (1,000 g for 15 minutes), and the supernatants were stored at −80°C until they were assayed. Serum concentrations of IL6 were determined by quantitative sandwich ELISA (Human IL6 Quantikine HS ELISA Kit, R&D Systems, Minneapolis, MN, USA), according to the instructions of the manufacturer.

### Statistical Analysis

The SNP rs484525 genotypes were tested for deviations from the Hardy-Weinberg equilibrium against controls using PLINK v1.07 (http://pngu.mgh.harvard.edu/~purcell/plink/index.shtml) and showed no significant deviation (p>0.05). Power analysis was carried out using the Power and Samples Size Program. The allelic and genotypic association of rs4845625 with AF was assessed using Pearson’s Χ^2^ test with a 2×2 and 2×3 contingency table (SPSS, version 13.0). Odds ratios (ORs) and 95% confidence intervals (CIs) were estimated using the Χ^2^ test (SPSS, version 13.0). Multivariate analysis was performed by incorporating age, sex, hypertension (HT), and diabetes as covariates and using multivariate logistic regression (SPSS, version 13.0). Observed P values were determined using PLINK v1.07. A linear regression model created with SPSS version 13.0 assessed the association between serum IL 6 levels and SNP genotypes.

## Results

### Patient Characteristics and AF Recurrence

In our study cohort, all subjects were from the GeneID database, which is populated with data from a Han Chinese population. In addition, all of the patients were diagnosed with AF and had undergone AF catheter ablation.

Before the end of the 4-week follow-up period, 119 patients experienced ERAF and 159 patients did not. Between 3 and 12 months post-ablation, no AF episodes were detected in the 66 patients who had ERAF, while 53 patients had an AF recurrence. Also between 3 and 12 months post-ablation, 19 of the patients who had no ERAF experienced an AF recurrence. After 12 months of follow-up, there were 72 patients who experienced LRAF and 206 patients who did not. ERAF (within the first 4 weeks) was observed in 42.8% of the patients, whereas LRAF (between 3 and 6 months) occurred in 25.9%. In total, 140 patients were observed to be recurrence-free in the 12 months of follow up. No significant differences in the baseline clinical characteristics or echocardiographic results were observed between patients with ERAF and LRAF. There were also no significant differences in ACE inhibitor/ARB or beta-blocker treatment between patients with or without ERAF and LRAF (Table 1).

### SNP rs4845625 and AF Recurrence

To analyze the association between the SNP rs4845625 and AF recurrences, we compared patients with ERAF (119 patients) to those without ERAF (159 patients), and we also compared patients with LRAF (72 patients) to those without LRAF (206 patients). The results of our analysis showed that the T allele of rs4845625 increased the risk of both ERAF (odds ratio [OR]: 1.84, 95% confidence interval [CI] from 1.31 to 2.59, p = 4.10×10^−4^) and LRAF (OR: 1.92, 95% CI from 1.30 to 2.81, p = 0.001) (Table 2). The association was significant after adjusting for age, sex, hypertension, and diabetes. After adjusting for these characteristics, in the patients with ERAF, p = 2.96×10^−3^, OR was 1.71, and 95% CI were from 1.20 to 2.38; in the LRAF cohort, p = 0.007, OR was 1.80, 95% CI were from 1.28 to 2.55.

**Table 2:** Allelic association of rs4845625 with AF Recurrence.

Cohorts	T Allele Frequency	Without Adjustment*		With Adjustment^†^	
		*P-obs*	OR (95% CI)	*P-adj*	OR (95% CI)
with ERAF vs. without ERAF	0.55/0.40	4.10×10^−4^	1.84 (1.31–2.59)	2.96×10^−3^	1.71 (1.20–2.38)
(119 vs.159)					
with ERAF vs. no recurrence	0.55/0.40	4.77×10^−4^	1.84 (1.31–2.59)	8.85×10^−3^	1.75 (1.23–2.45)
(119 vs. 140)					
with LRAF vs. without LRAF	0.58/0.42	0.001	1.92 (1.30–2.81)	0.007	1.80 (1.28–2.55)
(72 vs.206)					
with LRAF vs. no recurrence	0.58/0.40	3.36×10^−4^	2.10 (1.40–3.16)	3.51×10^−3^	1.90 (1.28–2.77)
(72 vs. 140)					

We also analyzed the association between rs4845625 and AF recurrence by comparing patients with ERAF (119 patients) to those patients who did not experience a recurrence (140 patients) and patients with LRAF (72 patients) to those who did not have a recurrence (140 patients). The results also showed that the T allele of rs4845625 increased the AF recurrence in our cohort (ERAF: observed p = 4.77×10^−4^ with an OR of 1.84 and adjusted p = 8.85×10^−3^ with an OR of 1.75, LRAF: observed p = 3.36×10^−4^ with an OR of 2.10 and adjusted p = 3.51×10^−3^ with an OR of 1.90).

The genotypic association of rs4845625 with AF was significant in both the ERAF cohort and the LRAF cohort (Table 3). In all three models (dominant, recessive and additive), we observed a higher recurrence rate in those patients who carry the T allele than those who carry the C allele.

**Table 3:** Genotypic Association of rs4845625 with AF Recurrence in Different Genetic Models.

	Model	AF Recurrence	Without Adjustment*		With Adjustment^†^	
			*P-obs*	OR (95% CI)	*P-adj*	OR (95% CI)
ERAF	Dominant		0.001	2.78 (1.54–5.02)	0.001	2.87 (1.57–5.21)
	CC (n = 74)	19 (25.7)				
	CT+TT (n = 204)	100 (49.2)				
	Recessive		0.02	2.08 (1.14–3.8)	0.02	2.18 (1.18–4.02)
	TT (n = 54)	31 (57.4)				
	CT+CC (n = 224)	88 (39.3)				
	Additive		0.001	n.a	0.01	2.05 (1.40–2.99)
	CC (n = 74)	19 (25.7)				
	CT (n = 150)	69 (46.0)				
	TT (n = 54)	31 (57.4)				
LRAF	Dominant		0.005	2.79 (1.35–5.80)	0.01	2.86 (1.37–5.98)
	CC (n = 74)	10 (13.5)				
	CT+TT (n = 204)	62 (30.4)				
	Recessive		0.006	2.39 (1.28–4.48)	0.02	2.44 (1.29–4.63)
	TT (n = 54)	22 (40.7)				
	CT+CC (n = 224)	50 (22.3)				
	Additive		0.002	n.a	0.001	2.11 (1.38–3.24)
	CC (n = 74)	10 (13.5)				
	CT (n = 150)	40 (26.7)				
	TT (n = 54)	22 (40.7)				

## Discussion

In this study, we analyzed the genotype of the *IL6R* gene for the SNP rs4845625 in 278 AF patients who experienced catheter ablation. By monitoring the recurrence of AF in these patients for 12 months, we were able to analyze the association of the rs4845625 genotype with AF recurrences after ablation. The results showed that the variant rs4845625 of the *IL6R* gene confers a significant risk of AF recurrence after catheter ablation in the Chinese Han population. The SNP rs4845625 was identified as an intronic SNP in the *IL6R* gene in association with AF risk before ablation. To the best of our knowledge, this is the first time that this variant in *IL6R* was shown to be associated with a risk of recurrence after catheter ablation.

The mechanism of AF is uncertain. Inflammation may play a role as a causative agent or as a marker to indicate AF risk [6]–[10]. Frustaci et al reported that atrial biopsies from 12 patients with lone AF were more likely to have inflammatory infiltrates, myocyte necrosis, and fibrosis, whereas biopsies from control patients were normal [8]. Marcus et al reported that CRP and IL6 levels were elevated in patients presenting with AFL (atrial flutter). The levels of CRP and IL6 fell significantly after the ablation of AFL and therefore atrial tachyarrhythmia appears to be the cause rather than an effect of inflammation [9]. Further, CRP and IL6 levels were significantly higher when blood was drawn from patients during AF compared to blood drawn from patients with normal sinus rhythm [10]. These data indicate that the inflammation pathway may affect the recurrence of AF after ablation.

Many studies have suggested that inflammation is an important mechanism in the pathogenesis of AF recurrence after catheter ablation. For instance, low CD36 levels in circulating monocytes and low serum hs-CRP levels were associated with AF recurrence after catheter ablation [11]–[14]. Elevated levels of IL6 were also shown to be associated with AF recurrence risk and were shown to be independent predictors of the recurrence of AF after an ablation [9], [12], [15], [16]. However, research as to whether the receptor for *IL6*, which conducts the inflammatory signal downstream, affects the recurrence of AF after ablation is limited. Our research in humans demonstrated that genetic variants in *IL6R* was associated with a recurrence of AF after ablation, and these results support the idea that inflammation, especially the IL6–IL6R pathway, has important effects on the risk of AF recurrence after ablation. Our research also suggested that anti-inflammation strategies may help prevent AF recurrence after ablation.

Previous studies showed that genetic factors can predict the risk of AF recurrence following catheter ablation. Family history was described as a risk factor for recurrent rehospitalization of patients with lone AF [17], and variants in 4q25, the angiotensin-converting enzyme (ACE) gene, CYP11B2 gene, and heme oxygenase-1 (HO-1) were shown to be associated with the recurrence of AF after catheter ablation [18], [19], [20]. In this study, we describe a novel genetic risk factor for the recurrence of AF after catheter ablation.

To test the possibility that the variant rs4845625 may affect the level of IL6, we assessed whether rs4845625 was associated with the mRNA expression and serum levels of IL6 in a healthy population. We first assessed whether rs4825625 affected the mRNA expression of IL6 using the public eQTL databases, such as Genevar and SNP Express, to determine the association of this SNP with mRNA expression in different tissues. We did not find any significant association between the genotype rs4845625 and the level of expression of IL6 mRNA. Because we did not have serum specimens for most of the subjects in our AF study, we randomly selected 16 patients of each genotype (CC, CT and TT) from the healthy population to measure their IL6 serum levels by ELISA. We did not find a significant difference in the IL6 level among the carriers of different genotypes (relative level of IL6 in serum: 0.53±0.19 for the CC genotype, 0.55±0.21 for the CT genotype and 0.52±0.24 for the TT genotype, P = 0.86 using the linear regression model).

To the best of our knowledge, the cohort of patients in our study of AF recurrence after ablation is one of the largest reported in China, however, the sample size is limited. A power analysis showed that our cohort can provide more than 70% power to detect an association, with an odds ratio (OR) of >1.53 for the early recurrence of atrial fibrillation cohort and an OR of >1.62 for the late recurrence of atrial fibrillation cohort (MAF = 0.48 according to HapMap CHB, type I error at 0.05). One limitation of the present study is that the sample size for both of the cohorts is underpowered when the power analysis assumes that the OR for the SNP rs4845625 in the Chinese population is from 1.53 to 1.62. Thus, we do not exclude the possibility that there is a falsely positive association; replication in larger cohorts should be performed.

The outcomes of our study were slightly different than those previously reported in other populations. In Germany, Kornej et al reported that ERAF was observed in 38% of patients within one week after ablation [21]. In our study, the ERAF rate (42.8%) was slightly higher. Two reasons may account for this difference. First, in our study, ERAF was defined as an AF episode during the first 4 weeks after the ablation, which is a longer observation time than previous studies. Second, compared to previous studies, we registered a higher proportion of patients with persistent AF (59.7%) and permanent AF (4.3%). In the studies from Kornej et al, all patients were diagnosed with paroxysmal AF. Furthermore, it has been reported that after ablation, 31% to 46% of patients are free of AF despite an early recurrence [22], which is in close agreement to what we have observed in this study. In our study, at the end of the follow-up period, 53 (44.5%) patients with ERAF still had AF, and 66 (55.5%) had no AF recurrence. At the end of the study, the total success rate of ablation was 74.1%.

This study included 278 patients and a low proportion of patients with permanent AF. It was reported that the effect of inflammation in different type of AF was various, our sample size is too small to draw conclusions for the relationship of inflammation with each type of AF, particularly with permanent AF. Some patients in our study had comorbid diseases, such as essential hypertension and diabetes. Patients with other structural heart diseases, such as rheumatic or non-rheumatic valvular heart disease, cardiomyopathy, and coronary heart disease, were excluded. Usually, the more severe the structural disease, the greater the severity and duration of inflammation will be. Consequently, the generalizability of our findings to other populations, such as patients with severe structural heart disease, is uncertain.

Rs4845625 is located in the intron of the *IL6R* gene; it is possible that the SNP rs4845625 serves as a marker for those patients at risk for AF recurrence after ablation and that the allele for a causative variant is in linkage disequilibrium with the SNP rs4845625. However, the genetic analysis in our study may indicate that the *IL6R* locus confers a risk for AF recurrence after ablation in the human population. Studies identifying the causal variant(s) with functional analyses may prove helpful.
